# Extraction of English Keyword Information Based on CAD Mesh Model

**DOI:** 10.1155/2022/2391898

**Published:** 2022-08-20

**Authors:** Xiuying Wu, Liuhui Yang

**Affiliations:** Department of Foreign Languages and General Studies, Shenyang Urban Construction University, Shenyang, Liaoning, China

## Abstract

Traditional methods only consider topic information in English vocabulary information extraction, lose the statistical feature information of the keywords themselves, and easily ignore the semantic information of the words. In order to improve the extraction efficiency of English keyword information, based on the CAD mesh model, this paper adds constraint factors such as vertex neighborhood flatness, vertex degree, side length, and flatness on both sides of the side on the basis of the original QEM quadratic error simplification algorithm, and it incorporates a smoothing effect into the edge folding cost function. Moreover, based on the proposed normal vector-based QEM mesh simplification algorithm, the point selection after the edge folding operation is fixed as the vertices of the original edge, and it is applied to the mesh parameterization. In addition, the algorithm solves the local parameterization problem of partially deleted vertices after the simplification operation of each layer is completed. After the model is constructed, the performance of the model is verified through experiments. The research shows that the English keyword information extraction model constructed in this paper is effective.

## 1. Introduction

Keyword extraction technology is a key technology in the field of text information mining. This paper takes a single document as the research object and proposes an algorithm for extracting keywords from English text based on a complex network [1]. Based on complex networks, the algorithm uses natural sentences as window units, words as nodes, co-occurrence relationship between words as edges, and word co-occurrence degree C (wxwy) based on the co-occurrence frequency as the weight of the edges to construct a text word co-occurrence network model based on a complex network [2]. Moreover, this paper constructs the comprehensive eigenvalue formula of nodes based on the degree centrality, eigenvector centrality, and node betweenness centrality of network nodes. The network nodes are output in descending order of comprehensive feature values, single word nodes are removed, and the first *K* words are extracted as text keywords [3].

The main task of automatic keyword extraction is to extract words that can express the main idea, main content, and author's opinion of the article from a specific article [4]. When we use a computer to extract keywords from a specific document, we can clearly understand the theme and main content of this document in a short time, so that we can obtain valuable information and improve our overall grasp of the content of this document [5]. The characteristic of English is that there is no obvious word boundary, and each word can appear independently. However, only by forming words with letters can we express specific meanings. If we want to get a single word, we need to perform English word segmentation first [6]. At the same time, texts in oriental traditional languages such as Japanese and Korean have the same characteristics. Word segmentation processing in these languages is also the key to intelligent text information technology. Currently, there are many word segmentation methods, and word segmentation algorithms are also different [7]. However, these methods are based on mechanical participle and comprehensive participle as the basic principles, and the final processing results are also different. English keywords are automatically extracted, and the process of English word segmentation cannot be avoided. At present, we still use English word segmentation technology for pre-processing [8]. However, English words are ever-changing, and different combinations of words and words have different meanings. This makes the processing of English word segmentation more complicated. Therefore, more manpower and material resources are needed to improve this work.

Keyword extraction algorithm based on statistics refers to an algorithm that uses the frequency of statistical words to perform related processing [9]. It judges whether a word is a keyword based on the statistical frequency of a word. Words and characters form a word. If a few consecutive words appear more often in the same statistical time period, they are likely to be a word. Therefore, the relationship between characters, such as frequency of occurrence, and the combination probability reflect whether they become a word [10].

## 2. Related Work

In order to further improve the effect and quality of keyword extraction, many scholars have improved the above algorithm. The literature proposed a text key phrase extraction method based on LDA and TextRank [11]. The literature uses a method that combines word position and word span to improve TFIDF weight, or uses word frequency and word position features to perform weighted analysis by using semantic coherence [12]. In addition, some scholars introduce the method of information entropy. However, these methods have certain problems in the application process, such as high computational complexity, and they need a considerable scale in terms of article type and corpus scale. There are also some scholars who combine article comprehensive information and quote news category factors, combined with other characteristic information for weighting, which can solve the problem of word frequency dependence to a certain extent [13]. However, these methods do not consider the impact of keywords' part of speech and keyword coverage. The literature proposed a method of keyword extraction based on graphs [14]. The method considers the context of the word, the position of the word, the centrality of the word, the part of speech, and other characteristics, and the initial weight of the word is modified. Meanwhile, it has obtained a good extraction effect. Keyword extraction methods based on relationship frequency have been widely used in foreign academic circles. Due to the characteristics of this method in the generation of horizontal networks, most applications eliminate the relationships that do not meet the threshold requirements and their corresponding keywords according to the set frequency (weight) threshold, and then expand the analysis based on the generated horizontal network [15].

## 3. The Theoretical Basis of Graph Theory of Plane Graph

**Figure 1:**
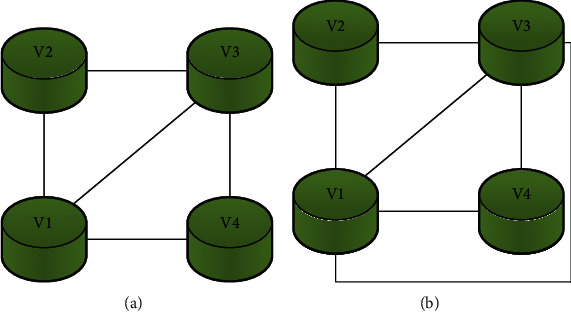
Plan embedding of graph. (a) Plane graph; (b) plan embedding graph.

If a graph *G* can be represented in a two-dimensional space, and the edges on the two-dimensional space do not intersect except for the common endpoints, the graph is a plane graph (plane embedding), denoted by *μ*(*G*). Graph *G* is called a planable graph. The floor plan divides the two-dimensional space into two regions, a bounded domain and an unbounded domain, and the boundary of the two domains (Jordan curve). As shown in Figure 1, Figure 1(a) is a plane graph, and Figure 1(b) is a plan embedding graph.

If the graph *G* = (*V*, *E*) is embedded in the *SR*^2^ plane to obtain *μ*(*G*), then the graph *G* is called the original graph of the plane graph *μ*(*G*). For mapping (immersion) *μ* : *G*↔*SR*^2^, *v* ∈ *V*, and *μ*(*v*) are regarded as interior points. The topological mapping (immersion) of (*u*, *v*) ∈ *E* is an edge (straight line) of *μ*(*G*). The boundary topology of the original image *G* is mapped to a Jordan line segment.

For any *e*^1^, *e*^2^ ∈ *E*, *μ*(*e*^1^) and *μ*(*e*^2^) simply intersect with individual common points. If *e*^1^ and *e*^2^ are not adjacent, they are recorded as $e^{1}\bar{adj}e^{2}$.

1. *μ*(*v*) : *G*↔*SR*^2^ is a bijection (one-to-one mapping);
2. *e*^1^, *e*^2^ ∈ *E*, $e^{1}\bar{adj}e^{2}↔\mu \left(e^{1}\right)\bar{a\;dj}\mu \left(e^{2}\right)$.

## 4. Theoretical Basis of Differential Geometry

According to the basis of differential geometry, the mapping from the plane domain *D*={(*u*, *v*)} to *R*^3^ is

(1) $$\begin{array}{c} r\left(u,v\right)=\left(x\left(u,v\right),y\left(u,v\right),z\left(u,v\right)\right). \end{array}$$

It satisfies

1. Each component (*x*, *y*, *z*) function is infinite order *C*^*n*^ continuous differentiable;
2. Vector

(2) $$\begin{array}{c} r_{u}=\left(\frac{\partial x}{\partial u},\frac{\partial y}{\partial u},\frac{\partial z}{\partial u}\right),\\ r_{v}=\left(\frac{\partial x}{\partial v},\frac{\partial y}{\partial v},\frac{\partial z}{\partial v}\right), \end{array}$$

are linearly independent, that is

(3) $$\begin{array}{c} r_{u}\wedge r_{v}\neq 0, \end{array}$$

where *r* is called a surface (patches) of *R*^3^ and (*u*, *v*) is called the parameters (coordinates) of the surface.

We assume that there is a surface *P* ∈ *R*^3^, its parameter representations is *r*=*r*(*u*, *v*), and the tangent vectors *r*_*u*_ and the tangent vector *r*_*v*_ form a tangent plane at any point on the surface P. The arbitrary tangent vector *η* on the surface P can be expressed as

(4) $$\begin{array}{c} \eta =\chi r_{u}+\beta r_{v}. \end{array}$$

The square of the length of the tangent vector *η* is expressed as

(5) $$\begin{array}{c} \langle \eta ,\eta \rangle =\chi^{2}\langle r_{u},r_{u}\rangle +2\chi \beta \langle r_{u},r_{v}\rangle +\beta^{2}\langle r_{v},r_{v}\rangle . \end{array}$$

Among them, we set

(6) $$\begin{array}{c} E=\langle r_{u},r_{u}\rangle ,F=\langle r_{u},r_{v}\rangle ,G=\langle r_{v},r_{v}\rangle , \end{array}$$

$\sqrt{E}$ is the length of the tangent vector *r*_*u*_, $\sqrt{G}$ is the length of the tangent vector *r*_*v*_, $F/\sqrt{EG}$ is the cosine of the angle formed by the tangent vector *r*_*u*_ and the tangent vector *r*_*v*_, and *g*=det*I*=*EG* − *F*^2^ is the metric tensor.

We assume that the parametric forms of P surface and $\tilde{p}$ surface are *γ*=*γ*(*u*, *v*), ((*u*, *v*) ∈ *D*) and $\tilde{\gamma }=\tilde{\gamma }\left(\tilde{u},\tilde{v}\right),\left(\left(\tilde{u},\tilde{v}\right)\in \tilde{D}\right)$, respectively, and the first basic row of the two surfaces are, respectively,

(7) $$\begin{array}{c} I\left(u,v\right)=Edu^{2}+2Fdudv+Gdv^{2}\\ \tilde{I}\left(\tilde{u},\tilde{v}\right)=\tilde{E}\tilde{d}{\tilde{u}}^{2}+2\tilde{F}d\tilde{u}d\tilde{v}+\tilde{G}d{\tilde{v}}^{2}\\ \text{d}p^{2}\left(u,v\right)=\text{d}{\tilde{p}}^{2}\left(\tilde{u},\tilde{v}\right)\\ \text{d}p^{2}\left[\text{d}u,\text{d}v\right]\left(\begin{array}{cc} E & F\\ F & G \end{array}\right)\left[\begin{array}{c} \text{d}u\\ \text{d}v \end{array}\right]\\ \text{d}{\tilde{p}}^{2}\left[\text{d}\tilde{u},\text{d}\tilde{v}\right]\left(\begin{array}{cc} \tilde{E} & \tilde{F}\\ \tilde{F} & \tilde{G} \end{array}\right)\left[\begin{array}{c} \text{d}\tilde{u}\\ \text{d}\tilde{v} \end{array}\right]. \end{array}$$

Under the corresponding mapping,

(8) $$\begin{array}{c} \left[d\tilde{u},d\tilde{v}\right]=\left[du,dv\right]\left(\begin{array}{cc} \frac{\partial \tilde{u}}{\partial u} & \frac{\partial \tilde{v}}{\partial u}\\ \frac{\partial \tilde{u}}{\partial v} & \frac{\partial \tilde{v}}{\partial v} \end{array}\right), \end{array}$$

is obtained and the existence of 1 : 1 mapping is a sufficient and necessary condition for conformal mapping:

(9) $$\begin{array}{c} \left[\begin{array}{cc} E & F\\ F & G \end{array}\right]=J_{\sigma }\left[\begin{array}{cc} \tilde{E} & \tilde{F}\\ \tilde{F} & \tilde{G} \end{array}\right]J_{\sigma }^{T}. \end{array}$$

Among them,

(10) $$\begin{array}{c} J_{\sigma }=\left(\begin{array}{cc} \frac{\partial \tilde{u}}{\partial u} & \frac{\partial \tilde{v}}{\partial u}\\ \frac{\partial \tilde{u}}{\partial v} & \frac{\partial \tilde{v}}{\partial v} \end{array}\right). \end{array}$$

Furthermore, we assume that *μ*=*χr*_*u*_+*βr*_*v*_ ∈ *T*_*P*_*S* is the tangent vector of the P surface at point *Q*, and set a curve of *P* with point *Q* as the starting point, namely

(11) $$\begin{array}{c} \gamma \left(t\right)=r\langle u\left(t\right),v\left(t\right)\rangle ,\\ \gamma \left(0\right)=P,{\frac{d\gamma }{dt}|}_{t=0}=r_{u}\frac{\text{d}u}{\text{d}t}\left(0\right)+r_{v}\frac{\text{d}v}{\text{d}t}\left(0\right)\\ =ar_{u}+br_{v}=\mu . \end{array}$$

Then, $\tilde{\gamma }\left(t\right)=\sigma \circ \gamma \left(t\right)$ is the curve on the surface $\tilde{P}$, and its tangent vector at *t*=0 is expressed as

(12) $$\begin{array}{c} \tilde{\mu }=\frac{\text{d}\tilde{\gamma }}{\text{d}t}\left(0\right)={\tilde{r}}_{u}\frac{\text{d}\tilde{u}}{\text{d}t}\left(0\right)+{\tilde{r}}_{v}\frac{\text{d}\tilde{v}}{\text{d}t}\left(0\right)\\ ={{\tilde{r}}_{u}\left(a\frac{\partial \tilde{u}}{\text{d}u}+b\frac{\partial \tilde{u}}{\text{d}v}\right)|}_{t=0}+{{\tilde{r}}_{v}\left(a\frac{\partial \tilde{v}}{\text{d}u}+b\frac{\partial \tilde{v}}{\text{d}v}\right)|}_{t=0}. \end{array}$$

The $\tilde{\mu }$ tangent vector depends on the mapping *σ* and the *P* surface tangent vector *μ*, so the mapping *σ*_*∗*_ between the tangent planes of the two surfaces can be derived from the mapping *σ* between the surface *P* and the surface $\tilde{P}$, which is also called the tangent mapping:

(13) $$\begin{array}{c} \sigma_{*}:T_{Q}P⟶T_{\sigma \left(Q\right)}\tilde{P}\\ \mu ⟶\tilde{\mu }=\sigma_{*}\left(\mu \right). \end{array}$$

In addition to the intrinsic properties of the surface, the triangular mesh model also relies on some other properties, of which the measurement of deformation is the most important. For a better understanding, we assume that *f*(*u*, *v*) exists on the surface. When an infinitesimal movement occurs at a point (*u*, *v*) in the parameter domain, the infinitesimal parameter displacement is defined as (Δ*u*, Δ*v*), and the first-order Taylor expansion on the surface corresponding to the new point *f*(Δ*u*+*u*, Δ*v*+*v*) is

(14) $$\begin{array}{c} \tilde{f}\left(\Delta u+u,\Delta v+v\right)=f\left(u,v\right)+f_{u}\left(u,v\right)\Delta u+f_{v}\left(u,v\right)\Delta v. \end{array}$$

**Figure 2:**
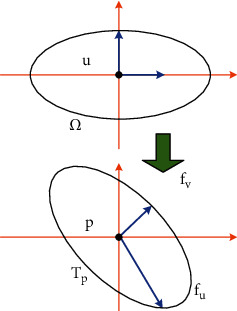
The first-order Taylor expansion $\tilde{f}$ of *f*.

The linear function maps all the vertices in the (*u*, *v*) neighborhood to the tangent plane *T*_*p*_ at the point *p*=*f*(*u*, *v*) ∈ *S*, and stretches the circle centered at (*u*, *v*) to an ellipse at the point *p* (as shown in Figure 2).

The expansion is rewritten as

(15) $$\begin{array}{c} \tilde{f}\left(u+\Delta u,v+\Delta v\right)=p+J_{f}\left(u,v\right)\left(\begin{array}{c} \Delta u\\ \Delta v \end{array}\right). \end{array}$$

Among them, *J*_*f*_(*u*, *v*) is the Jacobian matrix of *f*, that is, the 3 × 2 partial derivative matrix of *f*. Use singular value decomposition for the partial derivative matrix.

(16) $$\begin{array}{c} J_{f}=U\sum V^{T}=U\left[\begin{array}{cc} \sigma_{1} & 0\\ 0 & \sigma_{2}\\ 0 & 0 \end{array}\right]V^{T}. \end{array}$$

**Figure 3:**
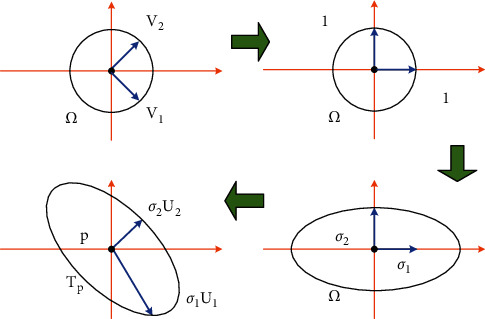
SVD decomposition of $\tilde{f}$.

Among them, the singular value is *σ*_1_ ≥ *σ*_2_ > 0, and the orthogonal matrix is *U* ∈ *R*^3×3^, and *V* ∈ *R*^2×2^. U, V decompose *U*_1_, *U*_2_, *U*_3_ and *V*_1_, *V*_2_ column vectors respectively. After that, the linear function $\tilde{f}$ is decomposed (as shown in Figure 3):

1. Deformation *V*^*T*^ first rotates all vertices around the center (*u*, *v*), so that the vector *V*_1_ and the vector *V*_2_ are located on the *u* v axis, and normalization is performed;
2. The deformation Σ is stretched at the ratio of *σ*_1_, *σ*_2_ on the *u* v axis;
3. Deformation U finally maps the two vectors to the tangent plane *T*_*p*_ at point *p.*

The circle with (*u*, *v*) as the center and radius *r* is mapped to an ellipse with point *p* as the center and semi-axis lengths *rσ*_1_ and *rσ*_2_, respectively, and orthogonal vectors *V*_1_, *V*_2_ are mapped to orthogonal vectors *σ*_1_*U*_1_, *σ*_2_*U*_2_, respectively.

The deformation of the circle stretched to an ellipse is called the local metric deformation of the triangular mesh model, which shows how the function *f* affects the parameter points of the (*u*, *v*) ∈ Ω neighborhood and the corresponding surface point *p*=*f*(*u*, *v*) ∈ *S*. All local metric deformation information is hidden between the singular values *σ*_1_, *σ*_2_.

  When *f* is isometric mapping or length-preserving mapping, ⇔*σ*_1_=*σ*_2_=1;
  When *f* is conformal mapping, ⇔*σ*_1_=*σ*_2_;
  When *f* is the area-preserving mapping, ⇔*σ*_1_*σ*_2_=1.

## 5. Merge Super Face

The general idea of the merge super-surface algorithm is to merge the adjacent triangular surfaces of the original model to form the so-called “super-surface.” In this way, one hole after another is formed on the surface of the original model, and then these holes are re-triangulated to obtain a new approximate model output. Compared with the original model, the new approximate model has fewer vertices and triangles, which can simplify the mesh.

The so-called hypersurface is a curved surface area composed of multiple polygonal surfaces, and the boundary of the curved surface is surrounded by the vertices of these polygonal surfaces, and the boundary of each polygonal surface forms a large non-planar polygon.

The algorithm has the following characteristics:

1. Within the given tolerance range, the output simplified mesh model is similar to the original mesh model. That is, the Euclidean distance between the vertex in the original mesh and the corresponding vertex of the output simplified model does not exceed the value *ε* set by the user. The opposite is the same, that is, the distance between the vertex of the simplified model and the vertex corresponding to the original mesh does not exceed *ε*.
2. The algorithm is very efficient. It is very practical to simplify very large grid models, such as those from medical CT and MRI data.
3. The topology information of the original mesh model is maintained.
4. The algorithm is domain-independent, it does not need to extract the original data to perform simplified operations. The vertex set of the hyperplane boundary is a proper subset of the vertex set of the original mesh model, so this algorithm is particularly suitable for the hierarchical representation of the original polygon mesh model.

The three steps of the grid simplification algorithm are as follows:

1. Create a hypersurface: The patch merging step mainly divides the patch set of the original model into hypersurface regions.
2. Boundary straightening: The boundary of the hyperplane area is simplified by merging boundary sets.
3. Triangulation of the hypersurface: After the triangulation of the hypersurface, the new vertex set is determined. In this step, a single hypersurface region will be decomposed into multiple hypersurfaces, and each sub-hypersurface has its own boundary and new triangulated vertices.

Among them, there are several rules for merging triangles:

(1) The rule of planarity: the distance from all vertices in the triangular face *f*_*b*_ to the plane *p* : *ax*+*by*+*z*=*d* must not exceed *ε*/2. That is, for
  (17) $$\begin{array}{c} v=\left(v_{x},v_{y},v_{z}\right)\in f_{b}, \end{array}$$
   there are
  (18) $$\begin{array}{c} \left|\left(a,b,1-d\right)\cdot \left(v_{z},v_{x},v_{y},1\right)\right|\leq \frac{\varepsilon }{2}. \end{array}$$
That is, a pair of linear constraints on (*a*, *b*, *d*):
  (19) $$\begin{array}{c} -\frac{\varepsilon }{2}-x_{z}\leq av_{x}+bv_{y}-d\leq \frac{\varepsilon }{2}-v_{z}. \end{array}$$
(2) Face axis rule: the direction of the triangular face *f*_*b*_ must be the same as the plane *p* : *ax*+*by*+*z*=*d*.
  (20) $$\begin{array}{c} \arccos\left(\frac{\left(a,b,1\right)\cdot \left(n_{x},n_{y},n_{z}\right)}{\sqrt{a^{2}+b^{2}+1}}\right)\leq \theta_{\max}. \end{array}$$
Among them, (*n*_*x*_, *n*_*y*_, *n*_*z*_) is the outward unit normal vector of the triangular face *f*_*b*_, and *θ*_max_ is the maximum angle between the two normal vectors.
(3) No folding rule: the triangular face *f*_*b*_ must not be folded on the super face.

**Figure 4:**
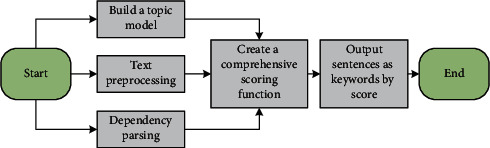
Flow chart of English text summary.

Hoppe proposed a series of edge shrinking operation mesh model simplification algorithm, as shown in Figure 4. This method records a series of detailed information and intermediate processes of edge contraction. After a period of iterative operation, the multi-resolution model (LOD) formed to meet the needs of different applications, and the algorithm is reversible, that is, the simplified final coarse mesh *M*^0^ can record information according to intermediate steps.

(21) $$\begin{array}{c} \left(\hat{M}=M^{n}\right)\overset{ecol_{n-1}}{⟶}\cdots \overset{ecol_{1}}{⟶}M^{1}\overset{ecol_{0}}{⟶}M^{0}. \end{array}$$

The original high-resolution mesh model *M*^*n*^ is obtained by performing the opposite point splitting operation, namely

(22) $$\begin{array}{c} M^{0}\overset{vsplit_{0}}{⟶}M^{1}\overset{vsplit_{1}}{⟶}\cdots \overset{vsplit_{n-1}}{⟶}\left(M^{n}=\hat{M}\right). \end{array}$$

## 6. Model Building

This article uses the local coherence model to calculate local coherence based on the relationship between adjacent sentences. First, this paper uses dependency syntax analysis to obtain the syntactic components of the words in the sentence, builds a text graph model with sentences as nodes based on this, and then calculates the local coherence of the sentences in the text. Secondly, this article uses the text word co-occurrence network model based on the complex network in the text keyword extraction described above to obtain the comprehensive feature value of the network node to meet the basic information requirements of the text in the text summarization process. Third, in order to improve the semantic information of the text, a text LDA model is constructed in units of sentences. By combining the three to construct a comprehensive scoring function, the sentences in the text are scored. Finally, this article sets a threshold (this article is limited to 10%), and selects text sentences within the threshold range as the text summary. The method flow is shown in Figure 4.

There are many keyword extraction algorithms. Common keyword extraction algorithms can be summarized into three categories: semantic-based algorithms, machine learning-based algorithms, and statistical model-based algorithms. Semantic-based keyword extraction algorithms rely on background knowledge bases, dictionaries, vocabularies, etc. Therefore, when extracting keywords from texts with many new online words and transliterated words, words or phrases that are not included in the knowledge base cannot be extracted. The keyword extraction algorithm based on machine learning relies on the selected algorithm model, and training takes a long time. The keyword extraction algorithm based on the statistical model has a simple principle, does not require training samples, does not rely on the knowledge base, and can extract words or phrases not included in the knowledge base. Aiming at the advantages and disadvantages of the above algorithms, this paper proposes a keyword group or phrase extraction algorithm based on the length of the common substring and the frequency of occurrence in the text under the English text. This algorithm is a keyword extraction algorithm based on statistical models. First, it extracts words or phrases that appear frequently in the text. A piece of English text is composed of multiple sentences, and the words or phrases with higher frequency in a piece of text can be extracted by extracting the common substrings of the sentences that make up the text. This method can extract words or phrases that appear twice or more in the text, and then combine the length and frequency of the extracted words or phrases to filter out keywords. This algorithm improves accuracy and does not require training samples and building models and can extract words or phrases that are not included in the knowledge base.

The English text is composed of sentences, so the English text can be processed by clauses to obtain the set of clauses corresponding to the text. Sentences are composed of words or phrases. Words or phrases are composed of characters. There are no spaces between words or phrases and the length is variable. Therefore, each clause can be regarded as a string of characters, and the two or two clauses can be improved. The common substring extraction algorithm can extract words or phrases that appear in both clauses, and finally get words or phrases with a frequency greater than or equal to two in the entire text. The length of the word or phrase extracted by this method is not limited, and the new words and transliterated words existing in the text can be accurately extracted without the limitation of the dictionary.

In this section, the improved longest common substring algorithm is used to extract words or phrases with higher frequency from short English texts. The main idea is to segment the short text and use the improved longest common substring algorithm to extract the words or phrases that appear in both clauses based on the obtained clauses, and to finally use the string matching method to calculate the frequency of the extracted words or phrases in the text, and filter out the words or phrases with a frequency greater than the threshold. This method extracts words or phrases that appear frequently in the text. The longer a word or phrase in an English text is, the more it has practical meaning and the more it can represent the theme of the text. Therefore, according to the frequency of the word or phrase in the text and the length of the word or phrase, the keywords in the word or phrase can be filtered out. In summary, the keyword extraction in this chapter is implemented based on statistical models, without training samples, nor relying on the knowledge base.

In the cluster center point selection algorithm based on the similarity between texts, how to select the cluster center point is also the difficulty of the algorithm after the similarity between the texts is obtained. Common methods for selecting clustering center points based on the similarity between texts include: selecting texts with less mutual similarity as the clustering center points, and selecting texts that are related to more texts as the clustering center Point, and selecting texts with greater similarity with part of the text as the cluster center point. Selecting the texts with less mutual similarity as the cluster center text: when the cluster center text selected by this method is used for clustering, the similarity between the clusters obtained is small. The disadvantage is that the randomness of the central text usually selected is relatively strong, and there may be “outliers.” Selecting the text that has correlation with more texts as the cluster center text: When the cluster center text selected by this method is used for clustering, the clustering result will not appear local optimal, and the clustering result is optimized. The disadvantage is that multiple cluster center points may belong to the same category, resulting in a category being divided into multiple categories. Select the text with greater similarity with part of the text as the cluster center text: when the cluster center text selected by this method is used for clustering, the clustering result will not appear locally optimal, and the clustering result is better. The disadvantage is that multiple clustering center points may also belong to the same category, resulting in a category being divided into multiple categories.

**Figure 5:**
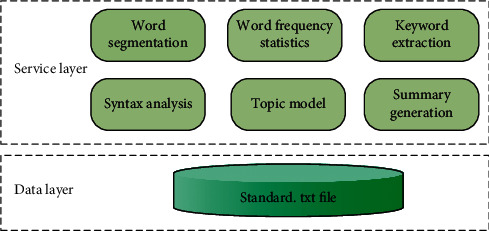
System software architecture.

In this paper, combined with actual R&D projects, a planning and design scheme of text platform software is proposed. The system architecture is shown in Figure 5.

**Figure 6:**
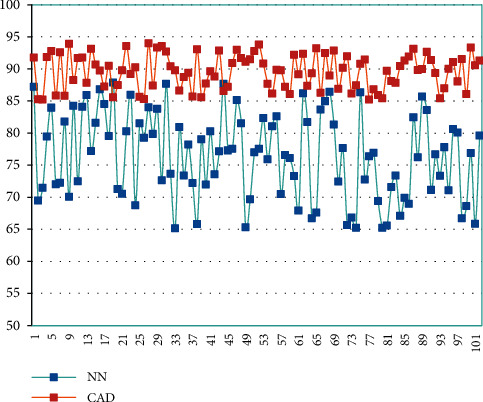
Comparison diagram of keyword extraction results.

Next, this article constructs an experiment to extract information from the English key words of the model. This article has set a total of 102 sets of texts. The keywords have been extracted by manual labeling. Therefore, the key word information can be extracted directly through the model proposed in this article, and the results can be compared with the manual labeling results. The model in this paper is named CAD, and the model in this paper is compared with the neural network model, and the neural network model is named NN. The keyword extraction results obtained on this basis are shown in Figure 6.

**Figure 7:**
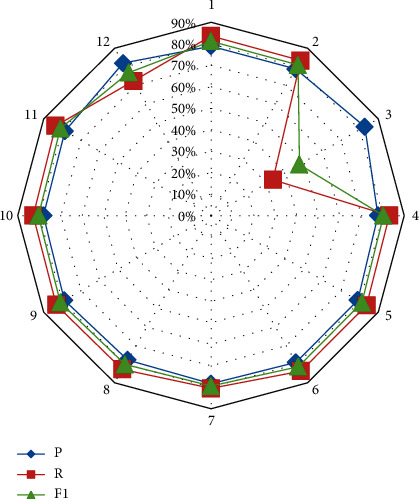
Statistical diagram of experimental results of feature analysis.

It can be seen from Figure 6 that the recognition rate of the model constructed in this paper can reach over 85%, and the highest can reach 95%, while the recognition rate of the neural network model is distributed between 65% and 85%. Next, in order to identify whether the 11 features proposed in the CAD model-based keyword extraction method can identify keywords, we conduct a feature analysis experiment. The experimental results of the feature analysis are shown in Table 1 and Figure 7.

From the above analysis results, we can see that the model constructed in this paper meets the expected requirements of the model constructed in this paper.

## 7. Conclusion

As one of the core technologies of natural language processing, keyword extraction from English text plays an important role in the application of natural language processing technology.

Mesh simplification and mesh parameterization are hot issues in the research of computer graphics. In recent years, rapid progress has been made in minimizing deformation energy and reducing the complexity of the algorithm itself. Based on the proposed normal vector-based QEM mesh simplification algorithm, this paper fixes the point selection after the edge folding operation to the vertex of the original edge, and uses it on the mesh parameterization. After the simplification operation of each layer of the grid is completed, the algorithm solves the local parameterization problem of partially deleted vertices, which avoids the complex calculations caused by solving large matrix systems. Successfully used in parametric-based heavy meshing and texture mapping applications. The experimental research results also prove that the model constructed in this paper has a certain effect.

## Figures and Tables

**Table 1:** Feature analysis experimental results.

	*P* (%)	*R* (%)	F1 (%)
1	78.92	83.65	81.21
2	78.77	83.39	81.01
3	82.67	33.35	47.53
4	77.80	82.98	80.31
5	78.92	83.66	81.21
6	79.02	83.65	81.27
7	78.37	80.56	79.45
8	77.74	82.71	80.14
9	78.94	83.24	81.03
10	78.06	82.84	80.39
11	78.63	83.65	81.06
12	81.96	72.36	76.86

## Data Availability

The data used to support the findings of this study are available from the corresponding author upon request.
